# Effect of particulate matter (PM_2.5_, PM_10_) on deaths and disability-adjusted life years (DALYs) in Gulf Cooperation Council countries: Global burden of disease time trend analysis 1990-2021

**DOI:** 10.12669/pjms.41.10.12523

**Published:** 2025-10

**Authors:** Sultan Ayoub Meo, Narmeen Shaikh, Anusha Sultan Meo

**Affiliations:** 1 Sultan Ayoub Meo, Department of Physiology, College of Medicine, King Saud University, Riyadh, Saudi Arabia; 2 Narmeen Shaikh, College of Medicine, King Saud University, Riyadh, Saudi Arabia; 3 Anusha Sultan Meo, The School of Medicine, Medical Sciences and Nutrition, University of Aberdeen, Scotland, United Kingdom

**Keywords:** Air Pollution, Chronic Obstructive Pulmonary Disease (COPD), Diabetes Mellitus, Gulf Cooperation Council (GCC), Ischemic Heart Disease, Particulate Matter (PM_2.5_, PM_10_)

## Abstract

**Objectives:**

Air pollution, especially particulate matter (PM_2.5_, PM_10_), is highly toxic and contributes to a range of diseases, resulting in premature morbidity and mortality. This study aimed to investigate the disease-specific mortality and disability rates attributable to air pollution in GCC countries from 1990 to 2021.

**Methodology:**

This time trend longitudinal study was conducted in the Department of Physiology, College of Medicine, King Saud University, Riyadh, Saudi Arabia, from July 2024 to December 2024. This study investigated the death and disability-adjusted life years (DALYS) rates of respiratory diseases, cardiovascular diseases, diabetes mellitus and total cancers caused by air pollution in the GCC region: Bahrain, Kuwait, Oman, Saudi Arabia, Qatar, and the United Arab Emirates. The data were collected from the Institute for Health Metrics and Evaluation (IHME) based on the Global Burden of Disease 2021 dataset from 1990 to 2021.

**Results:**

In GCC states, the highest rate of PM exposure in 2021 was in Qatar (56.95 per 100 people), whereas the lowest was in Oman (38.86 per 100 people). Qatar consistently achieved the highest decreases in death and DALY rates from 1990 to 2021. Among all the diseases, diabetes mellitus was the only one that presented an increase in deaths and DALYS in the GCC region from 1990 to 2021.

**Conclusion:**

The rising trend in air pollutant exposure is attributable to death and DALY rates in GCC countries. GCC states are at risk due to air pollution and associated with high mortality and DALY rates for different diseases.

## INTRODUCTION

Technological advancements in the modern era have contributed positively to the lifestyle of the masses; however, industrialization and urbanization have brought adverse effects due to air pollution.^1^ The increasing growth in emissions from vehicles, coal-fueled power plants, and the combustion of fuel oils and natural gas for heating all have contributed to the decline of air quality by releasing a various mixture of gases and particulates, such as ozone, noxious oxides, including carbon monoxide, nitrogen oxides, sulfur oxides, and fine particulate matter.^2^

Climate change and factors like temperature, wind patterns, precipitation, and the type and number of pollutants released all impact air quality.^3^ Acute and chronic exposure to air pollutants can lead to various adverse effects, including respiratory and cardiovascular problems^4^ and premature deaths.^5^

Particulate matter includes several tiny solid or liquid particles suspended in the air with a chemical composition of sulphate, nitrate, and mineral dust, among others.^6^ However, PM_2.5_, a particulate matter with a diameter of fewer than five micrometres, is considered highly toxic and a cause of various diseases owing to its tiny size. This allows it to be inhaled deep into the lungs and bloodstream in high concentrations, resulting in several disorders, premature death, and adverse birth outcomes.^7^

Air pollution continues to rise due to industrialization, the importance of our study’s findings becomes increasingly evident. The World Health Organization^8^ estimates that outdoor pollution was the cause of 4.2 million premature deaths worldwide in 2019, 89% of which occurred in low- and middle-income countries. In the Middle East, literature on air pollution is scarce, and further evidence is required to establish disease-specific causality with air pollution in terms of particulate matter. Our study, which analyses the rising particulate matter-specific air pollution trend in the GCC region from 1990 to 2021 and assesses the disease-specific mortality and disability rates due to air pollution, aims to bridge this literature gap and provide substantial evidence for a possible link for causality. The findings of this study will be invaluable in informing future policies and interventions to combat air pollution and its health effects.

## METHODOLOGY

This time trend longitudinal design study was conducted in the Department of Physiology, College of Medicine, King Saud University, Riyadh, Saudi Arabia, from July 2024 to December 2024. Focusing on the death rate and disability-adjusted life years (DALYs) rate of various diseases caused by air pollution from particulate matter, the study spanned from 1990 to 2021 in the GCC region, Bahrain, Kuwait, Oman, Saudi Arabia, Qatar, and the United Arab Emirates. We chose DALYs because they give a complete picture of the overall burden of disease by combining years of life lost due to premature mortality (YLLs) and years of life lost due to time lived in states of less than full health or years of healthy life lost due to disability (YLDs).^9^ We also extracted information about the exposure to particulate matter in each of the six countries within the region.

### Ethical approval:

The data were obtained from publicly available sources; therefore, the study was exempt from ethical approval or informed consent. IHME has been credited through proper citation in the methods and the reference sections.

### Data collection:

The data for this study were collected from the Institute for Health Metrics and Evaluation (IHME) website, a highly reputable source for global health data.^10^ We also selected the Global Burden of Disease 2021 study data. The website’s interactive tools enabled us to select the most relevant data for analyzing the mortality and DALYs of six specific diseases caused by air pollution. For this study, using the GBD estimate filter, we first extracted the data on particulate matter exposure per hundred population in each country for 1990 and 2021. Then, we changed the GBD estimate filter to risk factor and focused on the rate of occurrence as our metric and death and DALY as the indicators for which the estimates were produced. Air pollution, particulate matter, was chosen as our risk variable. We filtered all causes of death and DALYs and focused on the six diseases: ischemic heart disease, stroke, chronic obstructive pulmonary disease (COPD), diabetes mellitus (DM), respiratory infections and tuberculosis, and total cancers. Our filter included all age groups and both genders. We then selected a location that included all six GCC countries from 1990 to 2021.

### Statistical analysis:

The percentage change from 1990 to 2021 was calculated for DALYs and deaths caused by each disease due to air pollution in every GCC country. The formula used was percentage change = ((final estimate – initial estimate)/initial estimate) *100, where the final estimate was the 2021 reading and the initial estimate was the 1990 data for each variable. Mean, and standard deviation were calculated to get an average for the whole GCC region for each disease in 1990 and 2021 using SPSS version 29. Graphs were also constructed to depict the change in our variables over time.

## RESULTS

**Fig.1:**
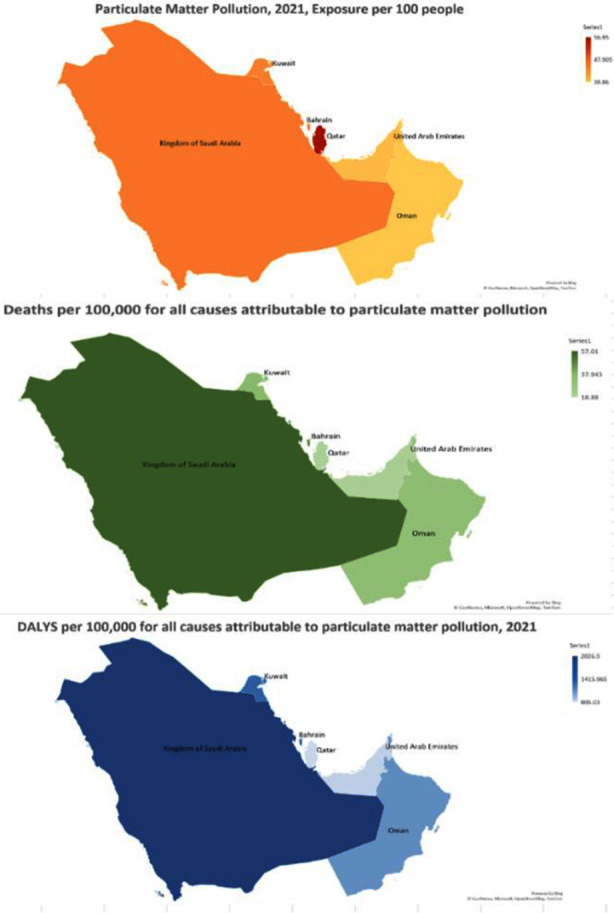
Country-wise depiction of particulate matter exposure per 100 deaths, and DALY rate per 100,000 for all causes attributable to PM pollution in 2021 in the GCC region.

The exposure of particulate matter (PM) per 100 in 1990 and 2021 and the % change between these two periods is shown in Table-I.. In all countries except Bahrain, PM exposure per 100 increased over the three decades. In Bahrain, there was a decrease of 1.23%, changing from 48.03 to 47.44 per 100 in 1990 to 2021. In the other countries, the highest increase was seen in Saudi Arabia, 23.21%, within the time frame. Focusing on the particulate exposure rates in 2021, the highest rate was in Qatar (56.95 per 100), whereas the lowest was in Oman (38.86 per 100). Fig.1 also shows us the geographical distribution of deaths and DALYs (per 100,000) for all causes due to particulate matter pollution in 2021, compared side by side with the particulate matter exposure (per 100) within each country for all ages and sexes.

**Table-I:** Particulate matter exposure per 100 population in 1990 and 2021 in the GCC region.

Country	1990	2021	% change
Bahrain	48.03	47.44	-1.23
Kuwait	42.60	44.61	4.72
Oman	37.94	38.86	2.42
Qatar	55.14	56.95	3.28
Saudi Arabia	36.28	44.70	23.21
United Arab Emirates	38.02	39.94	5.05

**Fig.2:**
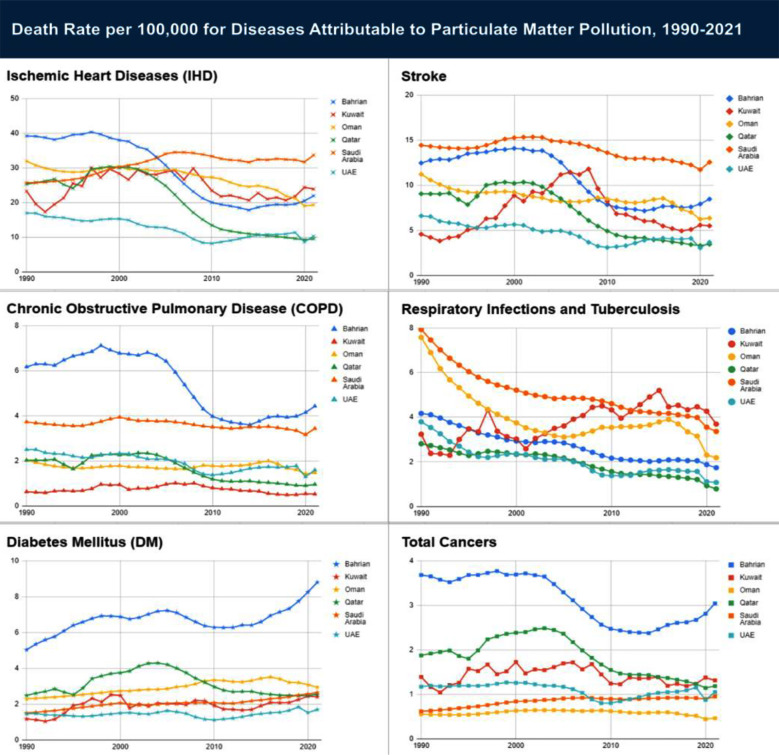
Death and DALY rates of diseases attributable to PM pollution between 1990 and 2021.

Table-II summarizes the mortality and DALYs for six diseases due to particulate matter pollution from 1990 to 2021 in Bahrain, Kuwait, Oman, Qatar, Saudi Arabia, and the United Arab Emirates. The results are presented as a rate per 100,000 population and a percentage change from 1990 to 2021. Fig.2 shows the trends in death rate per 100,000 for all ages and both sexes from 1990 to 2021.

**Table-II:** Deaths and DALYs for all causes and specific diseases due to particulate matter pollution from 1990 to 2021 in GCC countries.

Disease	Country	Deaths			DALYs		
		Rate per 100,000		% change	Rate per 100,000		% change
		1990	2021	1990-2021	1990	2021	1990-2021
All Causes	Bahrain	76.52	48.83	-36.19	2611.43	1557.06	-40.38
	Kuwait	36.65	37.96	3.57	1380.67	1316.07	-4.69
	Oman	64.87	33.88	-47.77	2632.46	1093.51	-58.46
	Qatar	47.98	18.88	-60.65	1829.21	805.03	-55.99
	Saudi Arabia	65.51	57.01	-12.98	2592.82	2026.90	-21.83
	UAE	37.28	19.64	-47.32	1569.30	830.72	-47.06
	GCC Mean rates ± SD	54.80 ± 16.56	36.03 ± 15.33	-----	2102.65 ± 576.25	1271.55 ± 468.56	-----
Ischemic Heart Diseases	Bahrain	39.18	21.93	-44.02	1066.01	599.87	-43.73
	Kuwait	23.28	23.86	2.49	673.89	656.61	-2.56
	Oman	31.95	19.31	-39.56	820.46	501.39	-38.89
	Qatar	25.29	9.52	-62.36	729.92	298.90	-59.05
	Saudi Arabia	25.65	33.64	31.15	680.95	1113.94	63.59
	UAE	16.94	10.27	-39.37	508.31	331.69	-34.75
	GCC Mean rates ± SD	27.04 ± 7.65	19.76 ± 9.05	-----	746.59 ± 186.62	583.73 ± 296.02	-----
Stroke	Bahrain	12.46	8.46	-32.10	332.30	227.98	-31.39
	Kuwait	4.57	5.50	20.35	144.47	158.33	9.59
	Oman	11.22	6.35	-43.40	301.39	181.62	-39.74
	Qatar	9.05	3.45	-61.88	289.56	134.54	-53.54
	Saudi Arabia	14.43	12.56	-12.96	359.38	401.70	11.78
	UAE	6.60	3.68	-44.24	211.81	149.70	-29.32
	GCC mean rates ± SD	9.72± 3.71	6.67± 3.43	-----	273.15 ± 80.40	208.98 ± 99.87	-----
Chronic Obstructive Pulmonary Disease	Bahrain	6.17	4.42	-28.36	169.10	138.70	-17.98
	Kuwait	0.63	0.52	-17.46	33.48	45.59	36.17
	Oman	2.03	1.47	-27.59	66.96	57.94	-13.47
	Qatar	2.02	0.95	-52.97	77.96	59.13	-24.15
	Saudi Arabia	3.72	3.43	-7.80	94.49	126.73	34.12
	UAE	2.50	1.59	36.40	94.23	88.65	-5.92
	GCC mean rates ± SD	2.85 ± 1.91	2.06 ± 1.52	-----	89.37 ± 45.09	86.12 ± 38.95	-----
Diabetes Mellitus	Bahrain	5.04	8.80	74.60	174.75	410.69	135.02
	Kuwait	1.18	2.42	105.08	82.43	256.90	209.12
	Oman	2.30	2.94	27.83	83.53	154.46	84.95
	Qatar	2.49	2.55	2.41	105.06	202.91	93.14
	Saudi Arabia	1.51	2.65	75.50	69.57	196.30	182.16
	UAE	1.47	1.70	15.65	65.21	163.45	150.65
	GCC mean rates ± SD	2.33 ± 1.42	3.51 ± 2.62	-----	96.76 ± 40.66	230.79 ± 95.26	-----
Respiratory Infection and Tuberculosis	Bahrain	4.16	1.74	-58.17	240.69	54.21	-77.48
	Kuwait	3.23	3.69	14.24	192.46	103.99	-45.97
	Oman	7.57	2.18	-71.20	495.80	79.70	-83.92
	Qatar	2.81	0.79	-71.89	168.04	31.44	-81.29
	Saudi Arabia	7.93	3.36	-57.63	481.99	120.53	-74.99
	UAE	3.79	1.07	-71.77	218.24	37.98	-82.60
	GCC mean rates ± SD	4.92 ± 2.25	2.14 ± 1.18	-----	299.54 ± 148.75	71.31 ± 36.20	-----
Total Cancers	Bahrain	3.68	3.04	-17.39	100.91	85.65	-15.12
	Kuwait	1.39	1.31	-5.76	40.23	35.73	-11.19
	Oman	O.56	0.47	-16.07	15.78	13.16	-16.60
	Qatar	1.87	1.19	-36.36	58.48	38.24	-34.61
	Saudi Arabia	0.62	0.95	53.23	17.40	29.68	70.57
	UAE	1.17	1.05	-10.26	38.24	34.55	-9.65
	GCC mean rates ± SD	1.55 ± 1.15	1.34 ± 0.88	-----	45.17 ± 31.60	39.50 ± 24.33	-----

In 2021, Saudi Arabia had the highest prevalence of deaths and DALYs of all causes due to PM pollution, 57.01 and 2026.90 per 100,000, respectively. Qatar had the lowest rate of deaths (18.88) and DALYs (805.03) in the same year. In contrast, Oman experienced the most significant decrease in all-cause DALYs due to PM from 1990 to 2021.

In 2021, Bahrain had the lowest death rate from ischemic heart disease due to PM pollution at 21.93 per 100,000 people, while Saudi Arabia had the highest death rate of 33.64. Bahrain, Oman, Qatar, and the UAE exhibited the highest reduction in their death rates in 2021 compared to 1990, and Qatar was in the lead with a decrease of 62%. Oman and Saudi Arabia have experienced increased rates over the past three decades. All countries have decreased DALY rates from 1990 to 2021, except Saudi Arabia, which reported a 63.59% rise. Overall, Saudi Arabia has seen an increase in both death rates and disability-adjusted life years (DALYs) for ischemic heart disease due to air pollution, but Qatar has experienced significant decreases in both metrics.

For stroke, Kuwait showed an increase in both the death rate (20.35%) and the DALYs (5.95%) from 1990 to 2021. All the other countries showed a decrease in death rate. The highest reduction in the death rate and DALYs was seen in Qatar, 61.885% and 53.54%, respectively. For Saudi Arabia, the death rate decreased, but the DALYs increased over the period. Saudi Arabia also reported the highest death rate (12.56 per 100,000) and DALY (401.7 per 100,000) in 2021 among all the GCC countries. Overall, the results demonstrated that the Saudi population was most affected by stroke caused by air pollution, whereas Qatar showed the most improvements over thirty-two years.

Saudi Arabia had the highest death rate and DALYs from chronic obstructive pulmonary disease (COPD) due to air pollution in 2021 at 14.43 and 126.73 per 100,000 people, respectively. On the other hand, Kuwait had the lowest death rate as well as DALYs at 0.63 and 45.59 per 100,000 people, respectively. From 1990 to 2021, the death rate was reduced in all countries except the UAE, which observed an increase of 36.4%. The DALYs were increased in Kuwait (36%) and Saudi Arabia (34%) between 1990 and 2021. Overall, Saudi Arabia stood out with an increase in DALYs, suggesting a growing burden despite a decrease in death rates.

Diabetes mellitus trends indicate a substantial rise in both death rates and DALYs across GCC countries, with Kuwait experiencing the most significant increases from 1990 to 2021. The death rate in Kuwait increased by 105.08%, while the DALYs increased by 209.12%. For 2021, the highest death rate was in Bahrain at 8.80 per 100,000, while the lowest was in the UAE with a rate of 1.70. The DALYs were also highest in Bahrain in 2021 at 410.69 per 100,000 and lowest in Oman at 154.46 per 100,000.

The death rates for respiratory infections and tuberculosis reduced in all countries except Kuwait from 1990 to 2021. Kuwait reported an increase of 14.24%. The lowest death rate was observed in Qatar in 2021 at 0.79 per 100,000 population. Kuwait reported the highest death rate in 2021 and was closely followed by Saudi Arabia. The DALYs were reduced in all countries from 1990 to 2021. The highest decrease was in Oman (83.92%), followed by the UAE (82.60%) and Qatar (81.29%). The highest DALY rate for respiratory infections in 2021 was seen in Saudi Arabia at 120.53 per 100,000 population.

The death rate for total cancers caused by PM pollution decreased in all countries except Saudi Arabia from 1990 to 2021, which reported an increase of 53.23%. Despite this increase, Saudi Arabia was not the country with the highest death rate in 2021. Bahrain had 3.04 total cancer deaths due to air pollution per 100,000 individuals, which was the highest rate among all GCC countries. The lowest percentage decrease was observed in Qatar (36.36%), while the lowest cancer death rate was in Oman at 0.47 per 100,000 people. DALYs decreased for all countries from 1990 to 2021, except in Saudi Arabia, where a 70.57% rise was noticed. The highest reduction was again in Qatar, with 34.61%.

Suppose we focus on the health impact trend of particulate matter among the regional population in 2021. In that case, ischemic heart disease (IHD) will take the lead by consistently reporting the highest death and DALY rates in all the countries. In 2021, the mean for the GCC region for IHD was 19.76 ± 9.05, while the DALY rate was 583.73 ± 296.02, both per 100,000 population and the highest reading among all other diseases. However, it’s worth mentioning that despite the elevated rate, the average decreased compared to 1990, when the death rate was 27.04 ± 7.65 and DALY was 746.59 ± 186.62. In contrast, deaths and DALYs due to air pollution’s impact on Diabetes Mellitus (DM) increased from 1990 to 2021. In 1990, the average deaths in the GCC region were 2.33 ± 1.42; in 2021, it rose to 3.51 ± 2.62 per 100,000 individuals. In 2021, the mean for the DALYs was 96.76 ± 40.66, which elevated to 230.79 ± 95.26 per 100,000. Air pollution had the lowest impact on total cancer in 2021, with the average rate being 1.34 ± 0.88 and 39.50 ± 24.33 per 100,000 for deaths and DALYs, respectively. For all causes, there was a general decrease in both deaths and DALYs, on average, over three decades. All the means and their standard deviations for deaths and DALYs for each disease in the entire GCC region have been summarized in Table-II.

## DISCUSSION

Air pollution, mainly due to particulate matter (PM), has emerged as a critical health issue worldwide, including in the GCC region. The GCC region’s rapid industrialization and urbanization have increased air pollutant levels in the past few decades. Our study focused on the health impact of PM from 1990 to 2021 in Bahrain, Kuwait, Oman, Qatar, Saudi Arabia and the United Arab Emirates (UAE). In this study, we explored trends in particulate matter exposure and the deaths and DALYs caused by PM exposure for all causes, specifically for six diseases of interest. Our findings revealed disparities in the impact of air pollution on mortality and DALYs for various diseases across the GCC countries. Qatar has been shown to have the highest levels of air pollution, with the highest PM exposure per 100 population. Despite this, Qatar has made remarkable progress in managing health conditions, demonstrated by the country consistently achieving the highest decreases in death and DALY rates for most diseases in 2021 compared to 1990. These findings highlight the potential for positive change in health and disease management despite high PM exposure.

On the other hand, other countries faced increasing challenges with specific diseases. We found that the rate of deaths and DALYs per 100,000 in Saudi Arabia for various diseases was amongst the highest in the region for the six diseases of interest, indicating a growing health burden due to air pollution in the region. A study conducted in Saudi Arabia revealed that the national average concentration of PM _2.5_ in the country witnessed a dramatic increase from 28 μg/m3 to 45 μg/m3, with a growth rate of 2.3 μg/m3/year between 1998 to 2018, with areas of high health risks expanding in size.^11^

PM had the most significant impact on deaths and DALY rates of ischemic heart diseases in GCC in 2021. Among all the diseases, diabetes mellitus was the only one that presented an increase in deaths and DALYs GCC regional average in 2021 compared to 1990. Particulate matter can be considered a risk factor for cardiovascular diseases (CVD) and stroke, both ischemic and haemorrhagic. Evidence suggests that long-term exposure to particulate matter, particularly PM_2.5_, may result in systemic inflammation, oxidative stress, autonomic nervous system activation and direct particle translocation.^12^ These initial pathways result in secondary paths that all lead to an increased risk of cardiovascular and cerebrovascular diseases, including ischemic heart disease and stroke.^13^

Alexeeff et al.^13^ reported that a 10-µg/m3 increase in long-term PM_2.5_ exposure led to a 23% increase in IHD mortality and a 24% rise in cerebrovascular death rate.^13^ Studies in Canada on PM_2.5_ and cardiovascular diseases have also shown possible associations.^14^ Another study from China also supported the increase in chronic IHD deaths due to elevated particulate matter exposure and reported that by reducing the PM_2.5_ and PM_10_ levels to those recommended by the World Health Organization air quality guidelines, we can prevent an estimated 6.16% and 4.30% of IHD, respectively.^15^ Regarding stroke and PM association, one study did a comprehensive review of 103 articles spanning over 28 countries and reported that increases in PM exposure were linked to stroke-related hospital admissions and mortality.^16^ A study done in Kuwait reported that PM_2.5_ were associated with an excess number of cases and elevated premature mortality rates for ischemic heart diseases and strokes.^17^

Diabetes Mellitus has been linked to PM pollution. Esposito et al.^18^ discussed various possible biological pathways for this association, including endothelial dysfunction resulting in decreased peripheral glucose uptake, inflammation leading to dysregulation of visceral adipose tissues, hepatic insulin resistance and other possible mechanisms that can increase insulin resistance.^18^ A global burden of disease collaboration study analyzed global data on air pollution and diabetes in 2019, concluding that 1/5th of the cases of Type-2 diabetes worldwide were due to PM_2.5_ pollution.^19^ Studies from Japan^20^ and Indonesia^21^ have also reported that PM_2.5_ was associated with the prevalence of diabetes. A survey from 121 United States communities reported that a 10 μg/m3 increase in PM_2.5_ was significantly associated with a rise in diabetic hospitalizations by 1.14%. Another US-based study found significant associations between PM_2.5_ and diabetes mortality.^22^ All these studies support our results similar to the other literature from the region of a study conducted in Saudi Arabia, where an association was seen in the rise in PM_2.5_ and metabolic syndromes, including Diabetes Mellitus.^23^

Respiratory disorders are the most common side effects of air pollution, notably PM pollution. Literature on the health hazards due to PM pollutants is vast, and a global meta-analysis study suggests that the most common one is respiratory disorders in the form of asthma and COPD, with the main culprits being PM_2.5_, PM_10_ and Nitrogen dioxide (NO2).^24^,^25^

Another meta-analysis showed a correlation between short-term exposure to PM_10_ and PM_2.5_, with all-cause mortality and between PM_2.5_ and PM_10_ and respiratory, cardiovascular, and cerebrovascular death rates.^26^ Local studies within the GCC region include PM as a significant source of pollution. Evidence from Kuwait in the GCC region shows that PM_2.5_ pollution has been attributed to higher risk levels of respiratory illnesses than cardiovascular diseases.^17^ This vast literature supports our study’s results of a rising trend in pollutant exposure and attributable death and DALY rates. Feng et al.^27^ reported that fine particulate matter (PM_2.5_) exposure may increase cancer risk. There is very low evidence for lung cancer, low certainty evidence for breast cancer, and moderate evidence for both liver and oesophageal cancers. Cheng et al.^28^ found an increased cancer mortality in females with air pollutants. It is essential to understand the role of the green environment on human wellbeing and combat against chronic debilitating diseases and deaths due to air pollution.^29^

Air pollution is a modifiable risk factor for all diseases, which is crucial for physicians to understand the pathophysiological mechanisms in clinical settings. The PM production is challenging to control due to its vast emission sources, ranging from population growth, urbanization and industrialization. We must reduce emissions and decrease exposure, especially in densely populated areas, as the health impact can be detrimental. For a developed region, such as the GCC, a potent risk factor that contributes to high mortality and DALY rates must be considered an alarming indicator of the need to mitigate the hazards poses to the local population.

### Study Strengths and Limitations:

The major strength of our study is that, to the best of our knowledge, this is a first-of-its-kind study that used the Global Burden of Disease 2021 data to explore the impact of particulate matter pollution on mortality and DALYs of different diseases within the GCC region. Since the data was taken from a well-reputed source, the accuracy of the results is reliable. The study has some limitations. Since we used a publicly available source, we could not control or comment on how the data was collected. Also, we could not verify the results of the GBD 2021 data with the local authorities. Additionally, due to the unavailability of complete data on the IHME website regarding specific parameters of air pollution, such as ozone and nitrogen dioxide as risk factors and their association with diseases of interest, we could only focus our study on particulate matter pollution. On the website, the data was only available till 2021, so data from 2022 to 2024 has not been reported.

## CONCLUSION

Air pollution is highly toxic and a leading global public health problem. The air pollutants, PM_2.5_ and PM_10_, are associated with deaths and disability-adjusted life years (DALYs) in the GCC region. Among ischemic heart diseases, stroke, chronic obstructive pulmonary disease, and respiratory infections, diabetes mellitus presented with an increase in deaths and DALYS in the GCC region from 1990 to 2021. Environmental and public health policymakers must implement strategies to keep the living areas and cities clean and green, thereby minimizing air pollution, deaths, and disabilities in the region.
